# Functional NMDA receptors are expressed by human pulmonary artery smooth muscle cells

**DOI:** 10.1038/s41598-021-87667-0

**Published:** 2021-04-15

**Authors:** Yi Na Dong, Fu-Chun Hsu, Cynthia J. Koziol-White, Victoria Stepanova, Joseph Jude, Andrei Gritsiuta, Ryan Rue, Rosalind Mott, Douglas A. Coulter, Reynold A. Panettieri, Vera P. Krymskaya, Hajime Takano, Elena A. Goncharova, Dmitry A. Goncharov, Douglas B. Cines, David R. Lynch

**Affiliations:** 1 Department of Pediatrics and Neurology, Perelman School of Medicine, The Children’s Hospital of Philadelphia, University of Pennsylvania, Philadelphia, PA 19104 USA; 2 Rutgers Institute for Translational Medicine & Science, Child Health Institute of New Jersey, Rutgers, The State University of New Jersey, New Brunswick, NJ 08901 USA; 3 Department of Pathology and Laboratory Medicine, University of Pennsylvania, Philadelphia, PA 19104 USA; 4 Division of Pulmonary, Allergy, and Critical Care Medicine, Department of Medicine, University of Pennsylvania, Philadelphia, PA 19104 USA; 5 Department of Bioengineering, School of Engineering and Applied Sciences, University of Pennsylvania, Philadelphia, PA 19104 USA; 6 Division of Pulmonary, Critical Care and Sleep Medicine, Department of Internal Medicine, University of California, Davis, Davis, CA USA

**Keywords:** Ligand-gated ion channels, Respiration

## Abstract

N-methyl-d-aspartate (NMDA) receptors are widely expressed in the central nervous system. However, their presence and function at extraneuronal sites is less well characterized. In the present study, we examined the expression of NMDA receptor subunit mRNA and protein in human pulmonary artery (HPA) by quantitative polymerase chain reaction (PCR), immunohistochemistry and immunoblotting. We demonstrate that both GluN1 and GluN2 subunit mRNAs are expressed in HPA. In addition, GluN1 and GluN2 (A–D) subunit proteins are expressed by human pulmonary artery smooth muscle cells (HPASMCs) in vitro and in vivo. These subunits localize on the surface of HPASMCs and form functional ion channels as evidenced by whole-cell patch-clamp electrophysiology and reduced phenylephrine-induced contractile responsiveness of human pulmonary artery by the NMDA receptor antagonist MK801 under hypoxic condition. HPASMCs also express high levels of serine racemase and vesicular glutamate transporter 1, suggesting a potential source of endogenous agonists for NMDA receptor activation. Our findings show HPASMCs express functional NMDA receptors in line with their effect on pulmonary vasoconstriction, and thereby suggest a novel therapeutic target for pharmacological modulations in settings associated with pulmonary vascular dysfunction.

## Introduction

N-methyl-d-Aspartate (NMDA) receptors are a subtype of ionotropic glutamate receptors essential for synaptic plasticity and memory formation^1,2^. Hyperactivity and hypofunction of NMDA receptors are implicated in the pathogenesis of neurological and psychiatric disorders, respectively^1–3^. Classical NMDA receptors are hetero-tetramers composed of two obligatory GluN1 subunits along with two GluN2 (2A–D) subunits^1,4^. Different combinations of GluN1 and GluN2 subunits endow NMDA receptors with distinct biophysical and pharmacological properties and association with intracellular signaling molecules^5^. The subunit composition of NMDA receptors is region specific and subject to developmental regulations that can modulate their sensitivity to stimulation by potential agonists and toxins^5,6^. NMDA receptors have higher permeability to calcium than to sodium and potassium, and greater sensitivity to extracellular magnesium blockade, thus requiring membrane depolarization to open with high probability^1,7^. Activation of NMDA receptors by glutamate and its co-agonists glycine or d-serine, acting at GluN2 and GluN1 subunits, respectively, triggers calcium influx, which mediates neurophysiological processes at low levels of activation, but causes neuropathological outcomes at high levels, e.g. in disorders such as stroke, Alzheimer’s disease and Huntington’s disease^1,2^.

While the expression and function of NMDA receptors in the nervous system have been studied extensively, their presence in non-neuronal tissues, including lung, is largely unexplored^8^. NMDA receptor subunits are expressed in all lung regions including trachea^9^. Recent study identified pulmonary airway smooth muscle cells as one cell type expressing NMDA receptors in lung^10^. Activation of NMDA receptors on these cells leads to calcium release and airway contraction^10^. Analogous to the effect on neurons, excessive activation of NMDA receptors in lung triggers acute nitric oxide-dependent injury^11,12^, suggesting NMDA receptor-mediated excitotoxicity exists outside the central nervous system. We previously demonstrated that NMDA receptor activation is linked to tissue plasminogen activator (tPA)-mediated inhibition of pulmonary arterial contractility and induction of vascular permeability^13,14^. However, whether and where functional NMDA receptors are expressed in pulmonary artery has not been established. In the present study, we examined the expression of NMDA receptor mRNA and protein in human pulmonary artery (HPA) using real-time reverse transcription-polymerase chain reaction (RT-PCR) and immunohistochemistry followed by analysis of receptor function using electrophysiology and contractile response.

## Results

### NMDA receptor expression by human pulmonary artery smooth muscle cells in vivo and in vitro

**Figure 1:**
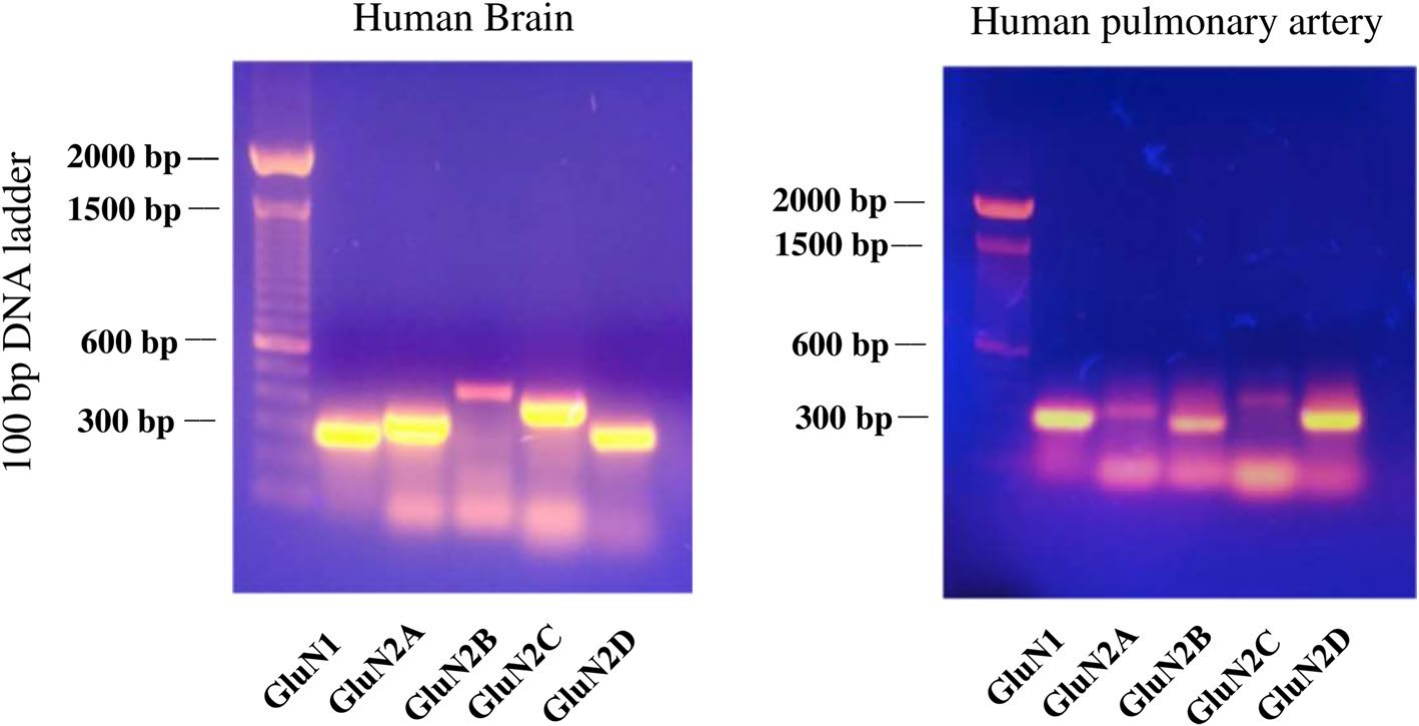
NMDA receptor mRNA expression in human pulmonary artery. RT-PCR was performed using RNA isolated from human pulmonary artery. The presence of mRNAs for GluN1 and all GluN2 subunits (2A–D) was detected in human pulmonary artery. Human brain RNA was used as a positive control.

To search for direct evidence of NMDA receptor expression within human pulmonary artery (HPA), we first performed RT-PCR using subunit-specific primers. Human brain was used as a positive control. mRNAs for both GluN1 and GluN2 subunits (GluN2A, GluN2B, GluN2C and GluN2D) were detected in HPA (Fig. 1). Of interest, the mobility of GluN2B cDNA differed between HPA (about 300 bp) (n = 3) and human brain (about 400 bp). Such difference was also detected in human pulmonary artery smooth muscle cells (HPASMCs) from a different donor (Supplementary Fig. S1). In addition, a primer targeting a different region^15^ of GluN2B amplified GluN2B mRNA in human brain but failed to amplify GluN2B mRNA in HPA (Data not shown). These results suggest that a splice variant of GluN2B is expressed in human pulmonary artery.

**Figure 2:**
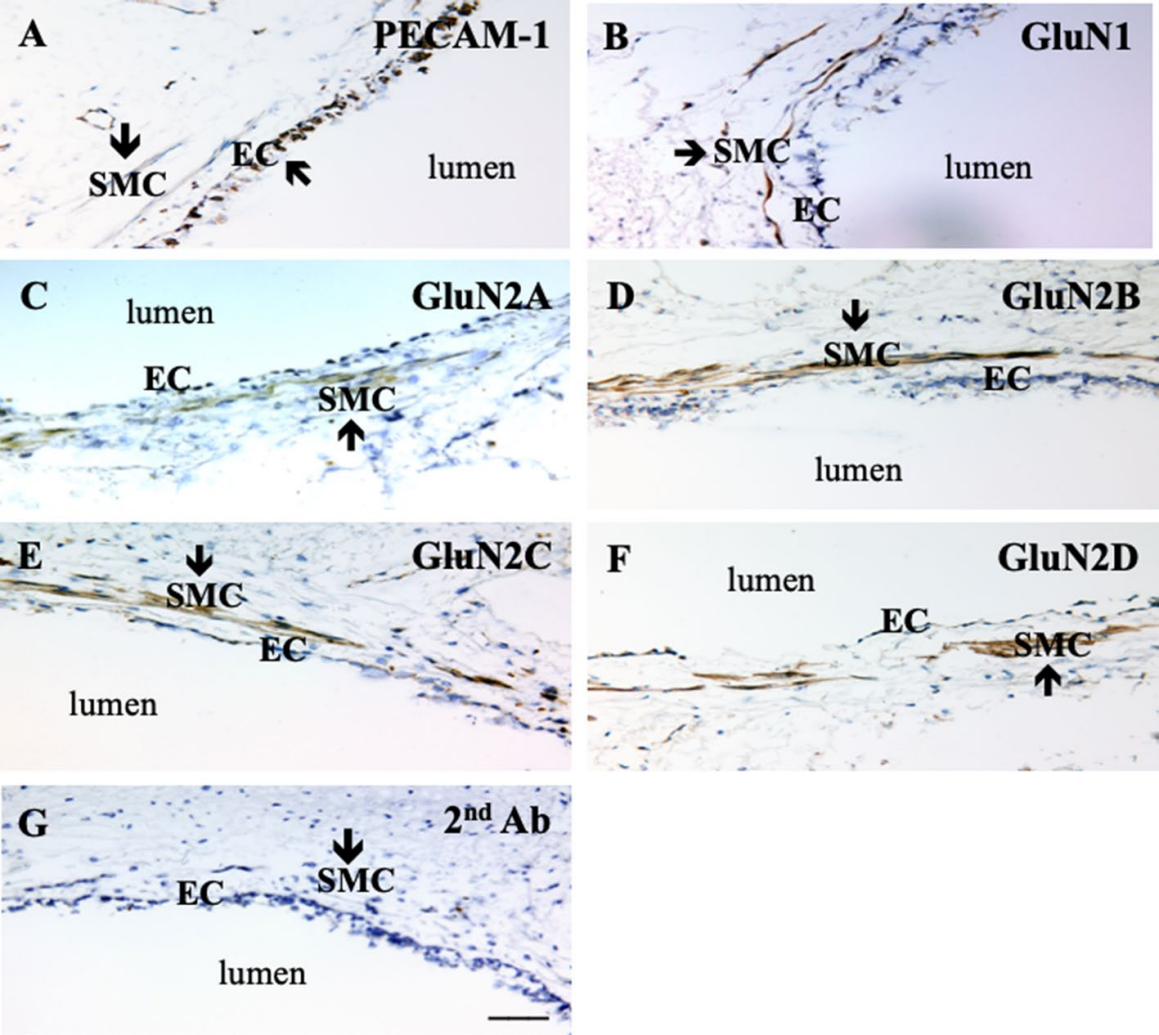
NMDA receptor expression by HPASMCs in vivo**.** Human lung tissue containing pulmonary artery was stained with NMDA receptor subunit specific antibodies. PECAM-1 was used as a marker for vascular endothelial cells (EC). Antibody against PECAM-1 revealed immunoreactivity within the interior surface of pulmonary artery. Immunoreactive staining for both GluN1 (**B**) and four GluN2 subunits (2A–D) (**C**–**F**) was observed in pulmonary artery smooth muscle cells (SMC). Control experiments with primary antibody omitted showed no immunoreactivity in either pulmonary artery endothelial cells or PASMCs (**G**). Scale bar = 50 μM.

We also performed immunohistochemistry on human lung tissue using validated NMDA receptor subunit-specific antibodies (Supplemental Fig. S2–S5). Antibody to PECAM-1 (Antibody validation: Supplemental Fig. S6), a marker of vascular endothelium, revealed intense staining of the interior surface of pulmonary artery (Fig. 2A). In contrast, immunostaining of GluN1 was associated with HPASMCs localized in smooth muscle cell layer (Fig. 2B). Strong immunoreactivity was also detected for all four subunits of GluN2 in HPASMCs (Fig. 2C–F), but not with secondary antibody alone (Fig. 2G). These results indicate that both GluN1 and GluN2 subunits are expressed in vivo by HPASMCs.

**Figure 3:**
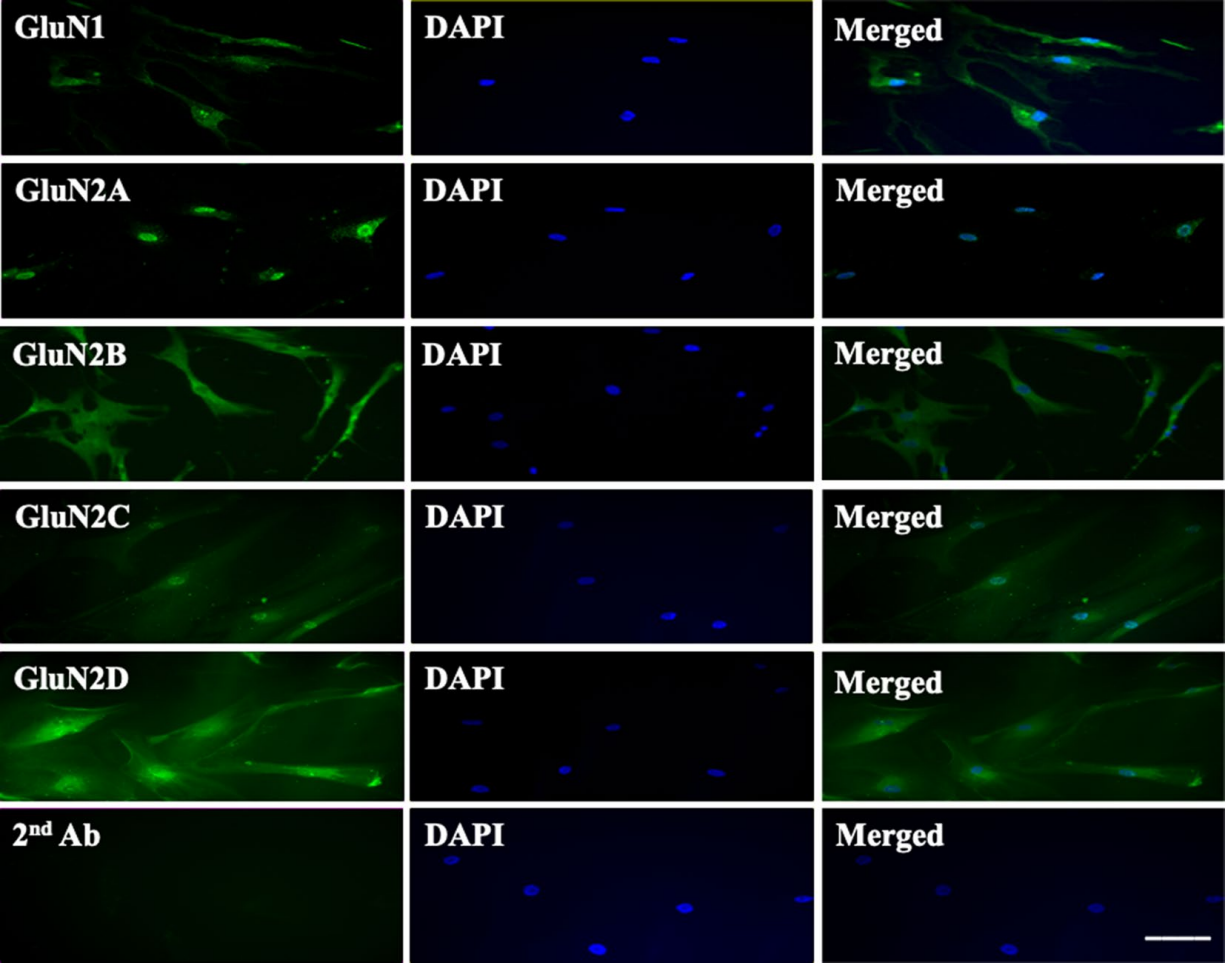
NMDA receptor expression in cultured HPASMCs. Immunofluorescence staining was performed on permeabilized HPASMCs. Network-like immunoreactive staining was observed for both GluN1 and all four GluN2 (2A–D) subunits. Control experiments with primary antibody omitted showed no immunoreactivity. Results are representative images taken from at least three separate experiments. Scale bar = 50 μM.

As an independent approach to study NMDA receptors expression, we performed immunofluorescence staining of cultured HPASMCs. Staining of permeabilized PASMCs demonstrated a network-like immunoreactivity for both GluN1 and GluN2 (A–D) subunits throughout the cell body (Fig. 3) (Antibody validation: Supplemental Figs. S4, S5, S7, S8). Immunoreactivity was not observed with secondary antibody alone (Fig. 3).

**Figure 4:**
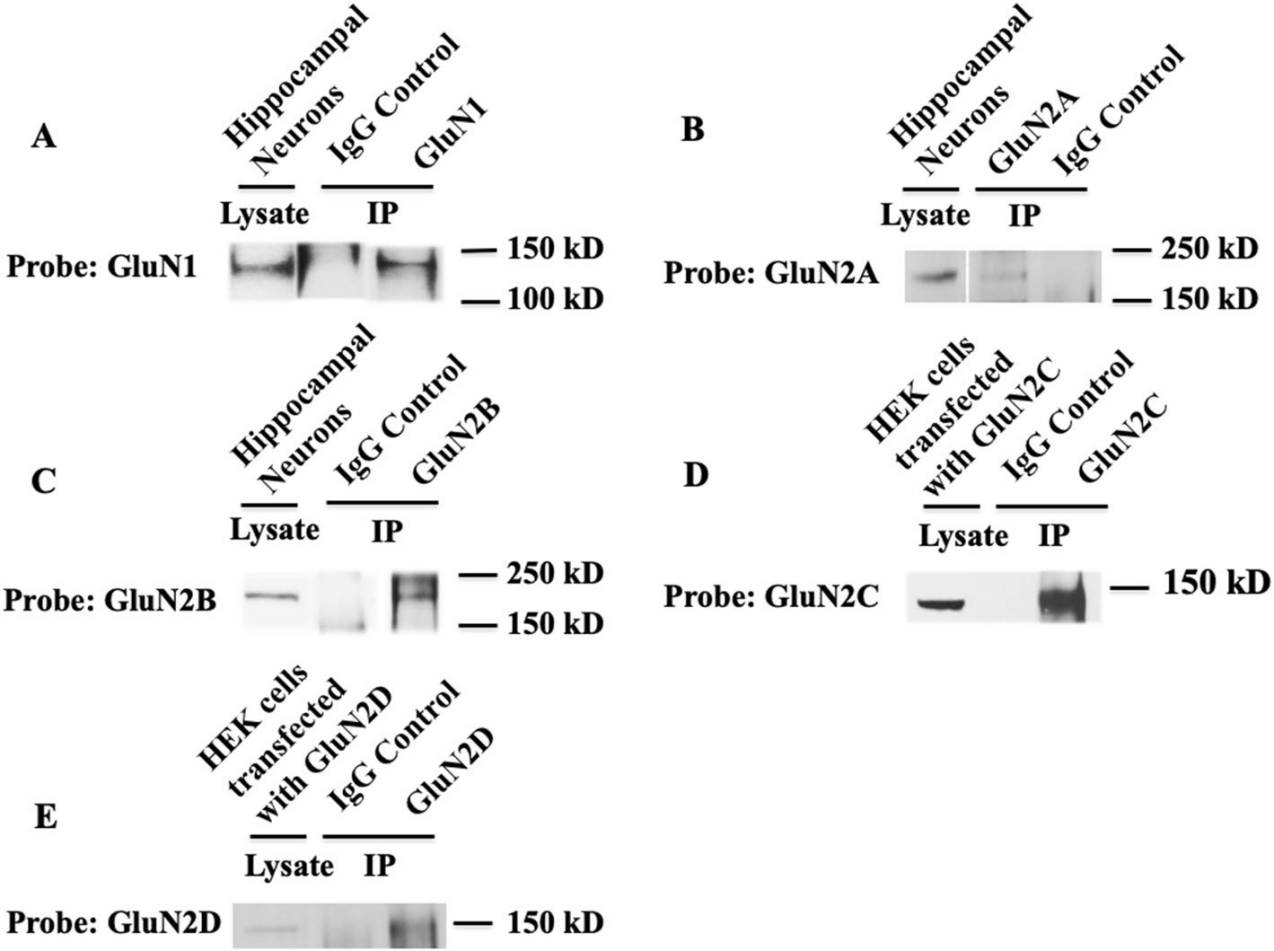
Full-length NMDA receptors are expressed by cultured HPASMCs. Cultured HPASMCs were lysed and immunoprecipitated with GluN1- or GluN2- subunit specific antibodies or control non-immune IgG, and the pellets were analyzed by Western blot. Immunopositive bands containing full-length GluN1 (**A**) and GluN2 (2A–D) (**B**–**E**) protein were detected in immune pellets from lysates of PASMCs but not in the IgG control conditions studied in parallel. Cropped images are shown for conciseness. Full-length blots are represented in Supplementary Fig. S9. Results are representative of three separate experiments.

To examine whether full length NMDA receptors are expressed, immunoprecipitation was performed on cultured HPASMCs with GluN1/GluN2 (A–D) subunit-specific antibodies. Full length GluN1 protein (120 kDa) was detected by immunoprecipitation using anti-GluN1 antibody to the intracellular domain (amino acids 660–811): the protein migrated at the same molecular weight as GluN1 protein from rat brain (Fig. 4A). No GluN1 immunoreactivity was detected with the IgG control (Fig. 4A). Similarly, addition of subunit-specific anti-GluN2A, anti-GluN2B, anti-GluN2C and anti-GluN2D antibodies immunoprecipitated GluN2A (MW = 180 kDa) (Fig. 4B), GluN2B (MW = 180 kDa) (Fig. 4C), GluN2C (MW = 140 kDa) (Fig. 4D) and GluN2D (MW = 150 kDa) (Fig. 4E), respectively, whereas no immunoreactivity was detected in the IgG control lanes. These results indicate that full length NMDA receptors are expressed by HPASMCs in culture.

### NMDA receptors on the surface of HPASMCs are functional

**Figure 5:**
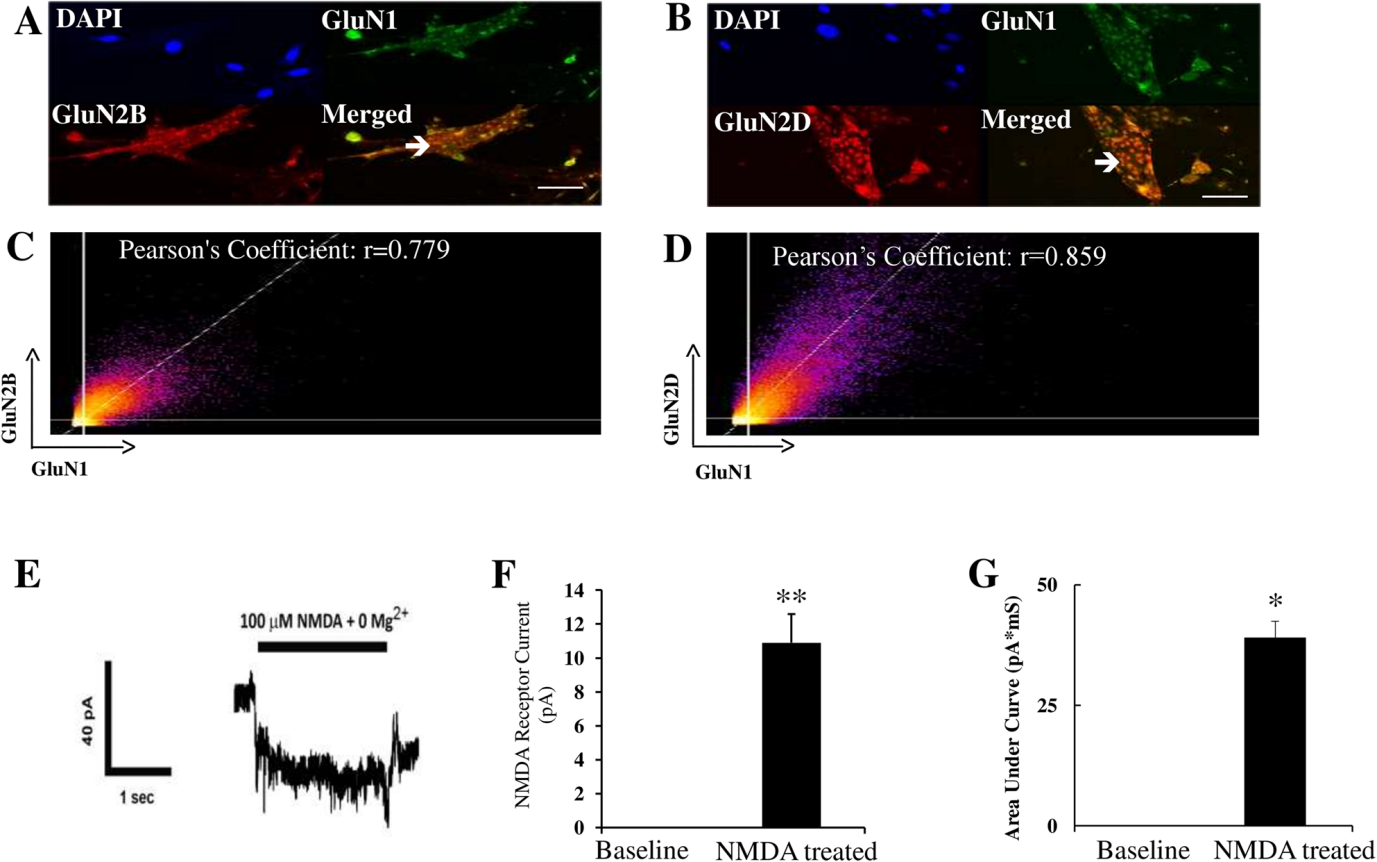
Functional NMDA receptors are localized on the surface of HPASMCs. Immunofluorescence staining was performed on non-permeabilized HPASMCs. Both GluN1 and GluN2 (2B and 2D) subunits were detected on the surface of HPASMCs (**A**,**B**). GluN1 co-localized with GluN2B (**A**) and GluN2D (**B**) on the surface of HPASMCs, respectively. Results are representative images taken from at least three separate experiments. Scale bar = 100 µM. (**C**,**D**) are representative scatter plots of each fluorescent pixel from confocal images with GluN1 green fluorescent intensity along the *x*-axis and GluN2 red fluorescent intensity along the *y*-axis. The shaded area in the upper right quadrant represents colocalized pixels above background with the associated Pearson correlation coefficient indicated for all colocalized pixels. (**E**) Representative traces for cultured HPASMCs in response to 100 μM NMDA and 10 μM glycine treatment. Cells were clamped at − 50 mV and whole cell recordings were performed. (**F**) NMDA and glycine treatment evoked an inward whole-cell current of 10.9 ± 1.7 pA (Mean ± SE, **p < 0.01, n = 16). (**G**) NMDA and glycine treatment evoked increased AUC compared with baseline (*p < 0.05, n = 16).

Co-expression of both GluN1 and GluN2 subunits on cell surface is required to form fully functional channels. Therefore, we next examined surface localization of NMDA receptors on the plasma membrane of unpermeabilized HPASMCs using antibodies against the amino-terminal extracellular epitope of GluN1 and GluN2 (B, D) subunits (Antibody validation: Supplemental Figs. S3, S5, S10). Punctate immunostaining for GluN1 and GluN2 (2B, 2D) was observed on the cell surfaces of HPASMCs (Fig. 5A,B). As only heteromeric complexes including both GluN1 and GluN2 subunit are targeted to plasma membrane, this finding suggests that GluN1 and GluN2 subunits co-localize on plasma membrane of HPASMCs. Consistent with this inference, the punctate appearance of surface-labeled GluN1 overlapped with that of surface-labeled GluN2B (Fig. 5A) and GluN2D (Fig. 5B), respectively. Quantitative analysis of the distribution of GluN1 and GluN2 (2B, 2D) demonstrated that GluN1 is highly associated with GluN2 subunits (Fig. 5C,D) (Pearson’s correlation coefficient: r = 0.779 and r = 0.859 for GluN2B and GluN2D, respectively), confirming that GluN1 and GluN2 subunits co-localize on the surface of HPASMCs.

We next assessed if NMDA receptors on the surface of cultured HPASMCs are functional, we used whole-cell patch-clamp electrophysiology to record currents evoked by the application of NMDA and glycine in a Mg-free recording solution. Application of 100 μM NMDA and 10 μM glycine to cells clamped at − 50 mV evoked an inward whole-cell current of 10.9 ± 1.7 pA (mean ± SE, *p* < 0.01 by t-test) (Fig. 5E,F) (n = 16). The area under curve (AUC) is increased by 39% over baseline with NMDA/glycine application (*p* < 0.05 by t-test) (Fig. 5G) (n = 16). To confirm this was mediated by NMDA receptor activation, we added the NMDA receptor antagonist AP5 (50 μM) to the cells. In the presence of AP5, application of NMDA and glycine failed to evoke an inward whole-cell current (n = 2) (data not shown), indicating this NMDA-evoked current in HPASMCs was mediated by NMDA receptor activation and that pulmonary arterial NMDA receptors are indeed functional. NMDA receptor currents in HPASMCs were small compared with NMDA receptor-evoked currents in rat hippocampal neurons (about 200 pA)^16,17^.

### Serine racemase and VGLUT1 expression in HPASMCs

**Figure 6:**
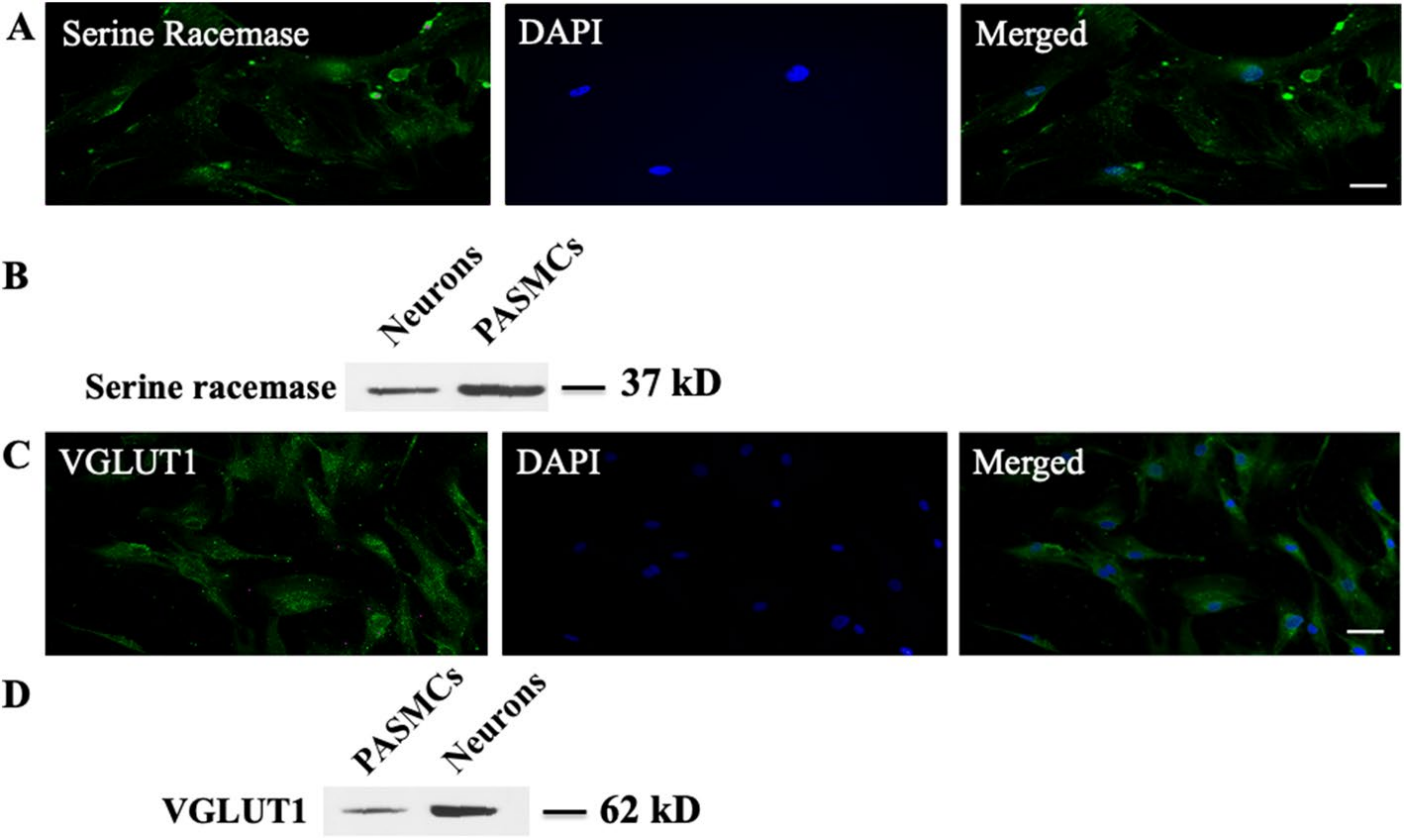
Serine racemase and VGLUT1 expression in HPASMCs. HPASMCs showed positive immunoreactive staining for serine racemase (**A**) and VGLUT1 (**C**), respectively. Immunopositive bands containing serine racemase and VGLUT1 protein were also detected in whole cell lysates from cultured HPASMCs ((**B**,**D**), respectively). Cropped images are shown for conciseness. Full-length blots are represented in Supplementary Fig. S13. Results are representative images taken from at least three separate experiments. Scale bar = 50 μM.

NMDA receptors are activated by glutamate and glycine, which bind to the GluN2 and GluN1 subunits, respectively. d-serine, another co-agonist acting at the glycine site, is generated from l-serine by serine racemase. To search for the potential endogenous co-agonist for NMDA receptor activation in HPASMCs, we stained cultured HPASMCs with an antibody against serine racemase (Antibody validation: Supplemental Fig. S11). Immunoreactivity for serine racemase was observed in HPASMCs (Fig. 6A). Serine racemase protein (37 kDa) was also detected on western blots of whole cell lysates from HPASMCs cultures (Fig. 6B) at the same molecular weight as serine racemase from rat brain neuronal lysates. This indicates that d-serine might be synthesized in HPASMCs and function as a potential local endogenous source for NMDA receptor activation.

We also stained cultured HPASMCs with antibody against vesicular glutamate transporter 1 (VGLUT1) (Antibody validation: Supplemental Fig. S12), which is responsible for vesicular storage of glutamate and subsequent release through exocytosis in both neuronal cells and endocrine cells^18,19^. Immunoreactive VGLUT1 was observed in HPASMCs (Fig. 6C). VGLUT1 protein (62 kDa) was also detected in whole cell lysates of HPASMCs (Fig. 6D), indicating that glutamate might be released from PASMCs and act with D-serine to activate NMDA receptors in an autocrine manner.

### NMDA receptors regulate contractility of pulmonary arteries in response to vasoconstrictor phenylephrine

**Figure 7:**
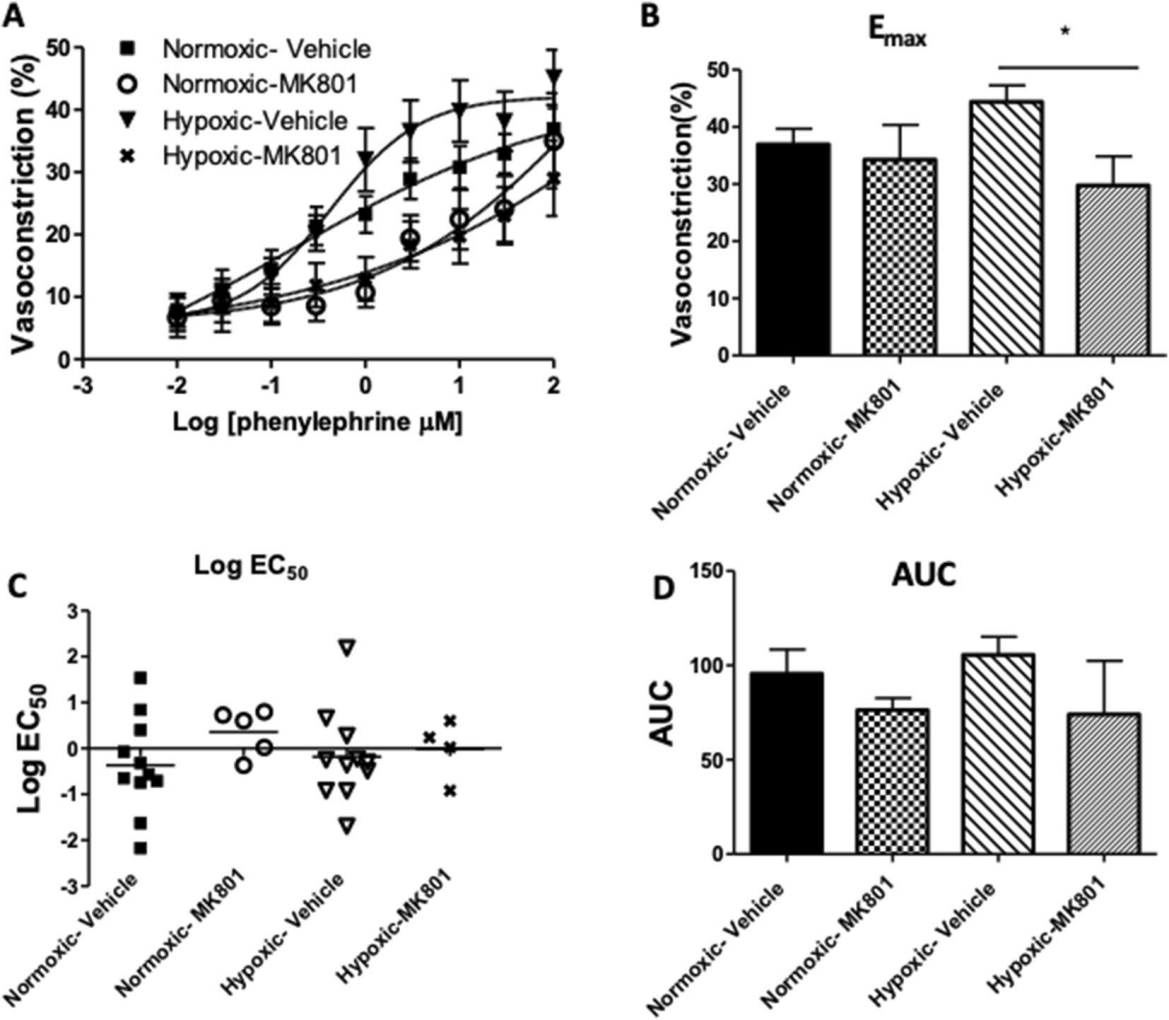
NMDA receptors regulate phenylephrine-induced pulmonary vasoconstriction. Precision-cut human lung slices containing pulmonary vessels were exposed to normoxic (21% O_2_) or hypoxic (5% O_2_) conditions overnight. The cells were pre-treated with 10 µM MK801 or vehicle (DMSO) for 1 h prior to adding varying concentrations of phenylephrine (PE), as indicated. (**A**) Phenylephrine induced pulmonary vasoconstriction in a dose-dependent manner in both normoxic and hypoxic conditions. (**B**) MK801 had little effect on maximal vasoconstriction (E_max_) under normoxic conditions, but significantly attenuated maximal response to phenylephrine under hypoxic conditions (*p = 0.0231). (**C**) MK801 had little effect on the sensitivity of the pulmonary vessels to phenylephrine-measured by Log EC_50_ of the dose–response curve-in both conditions. (**D**) MK801 had little effect on area under the curve (AUC) of the phenylephrine dose–response curve in both conditions. (Data are representative of: Normoxic-Vehicle, n = 13 donors; Normoxic-MK801, n = 6 donors; Hypoxic-Vehicle, n = 13 donors; Hypoxic-MK801, n = 6 donors).

Lastly, we investigated the functional significance of activating pulmonary arterial SMC NMDA receptors. We previously reported that the vasoreactivity of isolated pulmonary artery depends on activation of NMDA receptors^13,14^. In order to study the involvement of NMDA receptors in regulation of vasoreactivity of pulmonary vasculature in the context of the surrounding lung parenchyma, we measured the contraction of pulmonary arteries in human precision cut lung slices (hPCLS), as previously described^20,21^. Contraction was measured in response to the vasoconstrictor phenylephrine in the absence or presence of NMDA receptor antagonist MK801 (10 μM). Phenylephrine induced pulmonary arterial contraction in a dose-dependent manner under both normoxic and intermediate duration hypoxic conditions (Fig. 7A) (n = 13 for each condition). MK801 had little effect on maximal vasoconstriction under normoxic conditions, but significantly attenuated the maximal response to phenylephrine under intermediate duration hypoxic conditions (Fig. 7B) (n = 13 for both normoxic-vehicle and hypoxic-vehicle; n = 6 for both normoxic-MK801 and hypoxic-MK801). The sensitivity of the pulmonary vessels to phenylephrine-measured by Log EC 50 (Fig. 7C) and area under the curve (AUC) (Fig. 7D) were also measured in normoxic and intermediate duration hypoxic conditions. MK801 had little effect on these measurements in either conditions. These results suggest that NMDA receptors are involved in the regulation of pulmonary vasoreactivity in hypoxic conditions.

**Figure 8[replace with the revised figure]:**
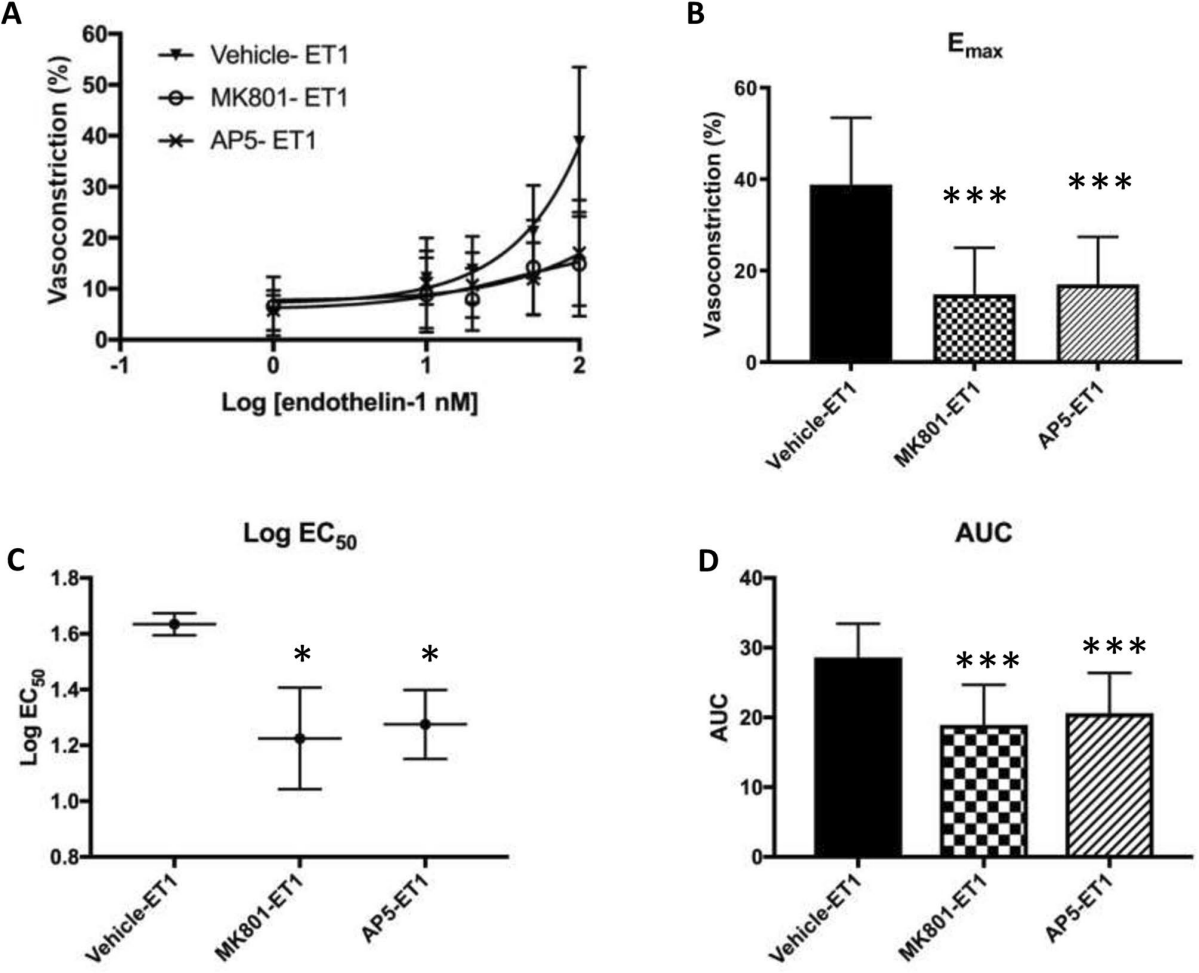
NMDA receptors regulate endothelin-1 (ET-1)-induced pulmonary vasoconstriction in murine precision-cut lung slices. ET-1 induced pulmonary vasoconstriction under a normoxic (21% O_2_) condition in a dose-dependent manner (**A**). Pretreatment with 10 µM MK801 or 50 µM AP5 significantly attenuated ET-1-induced maximal vasoconstriction (E_max_) (**B**), the sensitivity of the pulmonary vessels to ET-1 measured by Log EC_50_ of the dose–response curve (**C**), and the area under the curve (AUC) (D). *p < 0.05, ***p < 0.0001, n = 3.

To confirm the effect of NMDA receptors in pulmonary vasoconstriction, mouse precision cut lung slices were treated with endothelin-1 (ET-1) (10^–9^–10^–7^ M), a potent endogenous vasoconstrictor. While ET-1 initiated dose-dependent pulmonary vasoconstriction (Fig. 8A), both MK801 (10 μM) and AP5 (50 uM) significantly attenuated the maximal response to ET-1 (Fig. 8B), EC50 (Fig. 8C) and AUC (Fig. 8D) under normoxic condition (n = 3), confirming the involvement of NMDA receptors in the regulation of pulmonary vasoreactivity.

## Discussion

In the present study, we demonstrate that both GluN1 and each of the four GluN2 (2A–D) subunit proteins are expressed by HPASMCs. GluN1 and GluN2 subunits localize on the surface of HPASMCs and form functional receptors as evidenced by the whole-cell patch-clamp electrophysiology analysis performed in cultured HPASMCs (Fig. 5E–G) and by the difference in the maximal extent of contraction of pulmonary arteries measured ex vivo in hPCLSs in response to phenylephrine in the absence or presence of NMDA receptor antagonist MK801 under hypoxic conditions (Fig. 7). As NMDA receptor activation leads to the influx of calcium, which regulates vascular smooth muscle cell contractility, our results also suggest a potential role of NMDA receptor activation in pulmonary vasoconstriction under pathological conditions such as hypoxia.

The presence and activity of NMDA receptors in the lung has been demonstrated previously^9–12,22^. Excessive activation of NMDA receptors in the lung causes excitotoxicity leading to acute injury accompanied by disruption of pulmonary arterial contractility leading to increased permeability^11^, similar to findings in the CNS in which NMDA receptors-mediated excitotoxicity in neurons caused by pathologically high levels of glutamate leads to ischemia and neurodegeneration. Moreover, we previously reported that urokinase plasminogen activator, at pathological concentrations found in acute lung injury, inhibits pulmonary arterial contractility and promotes pulmonary vascular permeability through processes involving docking to NMDA receptors on pulmonary vascular smooth muscle cells^14^. Recent studies also point to the contribution of NMDA receptors in the pathophysiology of pulmonary arterial hypertension through mechanism involving HPASMC proliferation and migration^22,23^. The findings in this paper support these observations by providing physical evidence that functional NMDA receptors are expressed on pulmonary artery smooth muscle cells.

The precise combination of NMDA receptor GluN1 subunits with the various GluN2 subunits determines the biophysical and pharmacological properties of the NMDA receptors^24^. For example, GluN1/GluN2A heterodimers possess faster onset and shorter decay times than GluN1/GluN2B and GluN1/GluN2C channels, whereas GluN2D-containing NMDA receptors have the slowest decay times^24–26^. There is also evidence for the co-assembly of GluN2A/GluN2B, GluN2A/GluN2C, GluN2B/GluN2D, and other combinations in different regions of the brain and neuronal populations^24^, although the functional output and biophysical properties of these “mixed” channels are not understood in depth. GluN2 subunits are heterogeneously expressed in different regions of the brain. Here, we detected all four types of GluN2 subunits in pulmonary artery smooth muscle cells, implying the possible presence of multiple combinations of GluN1 and GluN2 subunits. Whether there is regional variation in subunit expression along th*e* vasculature and in disease states will require further study.

NMDA receptor function heterogeneity is determined by the C-terminal domain of GluN2 subunits interacting with various signaling proteins. The C-terminal domain is also subject to post-translation modification such as phosphorylation, which can potentiate NMDA receptor function^27,28^. The phosphorylation state also controls the internalization rate of NMDA receptors^29^, leading to altered NMDA receptor numbers on cell surfaces. Interestingly, hypoxia–ischemia in neonatal brain increases tyrosine phosphorylation of NMDA receptors, which correlates with enhanced association of Src protein-tyrosine kinase with NMDA receptors^30^. Whether such events contribute to the vasoconstriction under hypoxic conditions observed here remains to be investigated. Further investigations will also be needed to see if the presence of multiple GluN2 subunits in PASMCs diversifies the function of NMDA receptors through interactions with different signaling molecules, thus allowing pulmonary artery to be finely regulated in different physiological or pathological conditions.

Consistent with the previous reports that the localizations of d-serine and serine racemase are in close vicinity to NMDA receptors^31^, we found high levels of serine racemase in PASMCs, suggesting that d-serine could function as a co-agonist for NMDA receptors in PASMCs. As previously described^22^, we also observed expression of VGLUT1 in PASMCs, which confers a glutamatergic phenotype in neurons^19^. Previous findings have also identified VGLUT1 expression outside the brain in osteoblasts from which glutamate is released in a regulated manner^32^. The presence of VGLUT1 in PASMCs provides strong indirect evidence for presynaptic glutamatergic signaling events in PASMCs and also suggests that glutamate might mediate the intercellular communications between non-neuronal cells in an autocrine and/or paracrine manner. In addition to release from PASMCs^22^, glutamate in the blood stream may be taken up by pulmonary arterial endothelial cells (PAECs) through sodium-dependent or sodium-independent transporters^33^, from where it can be released and exert its action on NMDA receptors on adjacent PASMCs. Interestingly, hypoxia increases the rate of glutamate uptake by PAECs^33^. Hypoxic pulmonary vasoconstriction could arise from dysregulated glutamate release from PAECs and excessive NMDA receptor activation on PASMCs.

Deficiency of smooth muscle cell specific NMDA receptors attenuates hypoxic vascular remodeling and pulmonary hypertension through mechanisms involving decreased endothelial dysfunction, resistance to apoptosis and perivascular inflammation^22^. Our finding that NMDA receptor blockade reduces vasoconstriction under hypoxic conditions might suggest both a role in pulmonary hypertension and a beneficial effect of NMDA receptor blockade on ventilation/perfusion mismatch in acute hypoxia. The differential effects of NMDA receptor blockade on human and murine pulmonary vasoconstriction under normoxic conditions may signify species difference in NMDA receptor expression levels, post-translational modification and/or the extent of glutamate release. This inference may also be applicable to hypoxia^34–37^, as NMDA receptor blockade attenuates pulmonary vasoconstriction under hypoxia but has no effect under normoxia. In addition, hypoxia regulates endothelin-1, a potent vasoconstrictor released by endothelial cells^38,39^. Activation of endothelin-1 receptor increases calcium-dependent glutamate release from PASMCs and NMDA receptor phosphorylation in PASMCs^22^, highlighting the crosstalk between endothelin-1 receptor and NMDA receptor upon pulmonary vasoconstriction. NMDA receptor activation may represent a common pathway downstream of pulmonary vasoconstrictors. Future study will be needed to delineate signaling mechanisms between these receptors in health and disease states.

In summary, functional NMDA receptors are expressed by PASMCs. Activation of NMDA receptors can regulate pulmonary vasoconstriction. Characterization of number, distribution and regulation of pulmonary vascular NMDA receptors may provide insight into disorders associated with increased and impaired pulmonary vascular contractility, e.g. sepsis. Targeting NMDA receptor function may also represent a critical locus for preventing ventilation/perfusion mismatch, a site where acute lung injury is initiated in diverse settings and a potential locus for intervention to remediate dysregulation of vascular tone and permeability^40–42^.

## Methods

### Study approval

Postmortem human lung tissue was commercially obtained from the National Disease Research Interchange (NDRI, Philadelphia, PA) and the International Institute for the Advancement of Medicine (IIAM, Edison, NJ). The study protocol on human and murine lung tissue was approved by the Rutgers University Institutional Review Board and the Institutional Animal Care and Use Committee of the University of Pennsylvania, respectively. All experiments were performed in accordance with standard regulations.

### Real-time reverse transcription-polymerase chain reaction (RT-PCR)

HPA total RNA was purified using Qiagen’s RNeasy Mini kit (Qiagen, Germantown, MD) and then subject to RT-PCR using OneStep RT-PCR kit (Qiagen, Germantown, MD). 2 μg total RNA were used per reaction. Human brain RNA (Agilent, Cedar Creek, TX) was used as a positive control. The primer sequences for GluN2A, GluN2C and GluN2D were used as described^15^, and GluN1 and GluN2B primer sequences were designed using the NCBI Primer blast tool [https://www.ncbi.nlm.nih.gov/tools/primer-blast/]. The primer sequences were: GluN1 forward: 5′-CTACCCCCAACGACCACTTC-3′ and GluN1 reverse: 5′-TTCTCTGCCTTGGACTCACG-3′; GluN2B forward: 5′-GGACTGTCTCACCTTCTGCC-3′ and GluN2B reverse: 5′-TCTCTCTGTGCTGCCGTTG-3′. The primers were synthesized by Integrated DNA Technologies, Inc. (Skokie, IL).

### Antibody validation

Antibodies against GluN1, GluN2A, GluN2B or GluN2D were validated in HEK 293 cells transfected with plasmid DNA for GluN1, GluN2A, GluN2B or GluN2D, respectively using Lipofectamine™ 2000 reagent (Thermo Fisher Scientific, Hampton, NH)^43^. Either immunocytochemistry or Western blot (see below) was performed for antibody validation. GluN2C antibody was validated using human brain cerebellum whole tissue lysate (NB820-59180, Novus biological, Littleton, CO). Serine racemase antibody was validated using serine racemase knockout mouse-derived hippocampal lysate (leftover tissue from studies of Dr. Joseph T. Coyle at Harvard Medical School in Boston, MA).

### Cell culture

HPASMCs were obtained commercially (Lonza, Walkersville, MD) and cultured in Medium 231 supplemented with Smooth Muscle Growth Supplement (Life technologies, Grand Island, NY) at 37 °C under 5% CO_2_^44^. In all experiments, HPASMCs were studied before passage 6 prior to any change in cell morphology.

### Immunohistochemistry

Normal human lung tissue including pulmonary artery was cut into 5 μm sections and then mounted onto slides. Immunohistochemical staining was performed as previously described^45^. Briefly, sections were fixed and air dried followed by one wash in Tris-buffered saline (TBS). Endogenous peroxidase activity was quenched by incubating sections in 0.5% H_2_O_2_ in 30% methanol. After 3 washes in TBS, sections were permeabilized and pre-blocked followed by primary antibody incubation at the following concentrations: platelet endothelial cell adhesion molecule (PECAM-1) (ab28364, Abcam, Cambridge, MA) (1/150), GluN1 (556308, BD Pharmingen, San Jose, CA) (1/1000)^46^ (this reference contains characterization of the antibody against GluN1), GluN2A (AGC-002, Alomone, Jerusalem, Israel) (1/20,000), GluN2B (AGC-003, Alomone, Jerusalem, Israel) (1/3000), GluN2C (ab105146, Abcam, Cambridge, MA) (1/30,000) or GluN2D (ab186816, Abcam, Cambridge, MA) (1/10,000). After 24 h incubation at 4 °C, sections were washed for three times in TBS. Biotinylated secondary antibodies (Vector laboratories, Burlingame, CA) were then introduced for one hour at RT followed by avidin–biotin–horseradish peroxidase complex (Vector laboratories, Burlingame, CA) treatment for one hour at RT. The signal was developed with diaminobenzidine (Vector laboratories, Burlingame, CA). Photomicrographs were taken under brightfield with a Leica DM 4000B microscope.

### Immunocytochemistry

Human pulmonary artery smooth muscle cells (PASMCs) (Lonza, Walkersville, MD) were seeded on coverslips coated with Poly-d-Lysine (0.5 μg/ml) followed by fixation, permeabilization, blocking and antibody incubation as previously described^43^. The following primary antibodies were used for antigen labeling: antibody to GluN1 (556308, BD Pharmingen, San Jose, CA) (1/250), GluN2A (MAB5216, EMD Millipore, Burlington, MA) (1/100), GluN2B (71-8600, Thermo Fisher Scientific, Carlsbad, CA) (1/100), GluN2C (ab105146, Abcam, Cambridge, MA) (1/300), GluN2D (ab186816, Abcam, Cambridge, MA) (1/100), serine racemase (ab45434, Abcam, Cambridge, MA) (1/500), or VGLUT1 antibody (75-066 Neuromab, Davis, CA) (1/100). Fluorescent images were captured using Leica DM 4000B microscope.

For surface labeling of NMDA receptors, cells were fixed in 4% paraformaldehyde (PFA) (10 min, RT) and then blocked with 10% BSA for one hour at 37 °C^43^. Primary antibodies against GluN1 (Immunogen: N-terminal 42–361 amino acid) (SAB5200546, Sigma, Saint Louis, MO) and GluN2B (extracellular domain) (AGC-003, Alomone, Jerusalem, Israel) or GluN2D (N-terminal 400–500 amino acid) (ab186816, Abcam, Cambridge, MA) were added simultaneously and incubated overnight at 4 °C. After three washes in PBS, cells were incubated with Alexa Fluor 488^®^ and Alexa Fluor^®^ 568 conjugated secondary antibodies (Life Technology, Eugene, OR) for 1 h in the dark. Olympus FluoView laser scanning confocal microscope was used to capture fluorescent images which was analyzed using Image J software.

### Immunoprecipitation

Cultured HPASMCs were lysed in RIPA buffer^43^ followed by Immunoprecipitation as described previously^43^. The following antibodies were used: anti-GluN1 antibody (556308, BD Pharmingen, San Jose, CA), anti-GluN2A (AGC-002, Alomone, Jerusalem, Israel), anti-GluN2B (AGC-003, Alomone, Jerusalem, Israel), anti-GluN2C (ab105146, Abcam, Cambridge, MA) or anti-GluN2D (ab186816, Abcam, Cambridge, MA).

### Western blot

Western blot was carried out as previously described^43^. The following antibodies were used: mouse anti-serine racemase antibody (ab45434, Abcam, Cambridge, MA) and mouse anti-VGLUT1 antibody (75-066, Neuromab, Davis, CA).

### Whole-cell patch-clamp recording

In order to examine the NMDA receptor function, a whole cell voltage clamp technique was used to record the NMDA elicited current in cultured HPSMCs grown on coverslips^47,48^. Experiments were conducted at room temperature (23 ± 2 °C) in HEPES solution containing (in mM) 155 NaCl, 3 KCl, 3 CaCl_2_, 10 HEPES, pH 7.4. HPASMCs were voltage-clamped at a holding potential of − 50 mV to increase the driving force using a recording pipette with intrapipette solution containing (in mM) Trizma phosphate (dibasic) 100, Trizma base 28, EGTA 11, MgCl_2_ 2, CaCl_2_ 0.5 and Mg-ATP 10, pH 7.35, 290 mOsm. Recording signals were amplified using an Axopatch-1D amplifier (Axon Instruments/Molecular Device Corporation Sunnyvale, CA) and filtered at 5 kHz and then saved using pCLAMP 9.01 software (Axon Instruments, Inc. Foster City, CA) for off-line data analysis. Fast solution change time (< 3 ms) was accomplished via the combination of a solenoid-activated system and a step-perfusion device (Warner Instruments, Hamden, CT). 100 μM NMDA and 10 μM glycine (Sigma, St. Louis, MO) were applied to the cell for 2 s. Current amplitudes were calculated using Clampfit software (pCLAMP 9.01, Axon Instruments, Inc. Foster City, CA). Recordings were made from 16 cells from 3 donors. Statistical significance (*p* < 0.05) was determined using unpaired Student’s t-test when comparing different treatment groups.

### Human precision cut lung slices preparation and measurement of vasoconstriction

Human precision cut lung slices (hPCLS) were prepared and vasoconstriction was measured as previously described^21^, with modifications for vessels (instead of airways). Briefly, whole human lungs from 26 non-diseased donors (Supplemental Table S1 including all the donor information) were inflated using 2% (wt/vol) low melting point agarose and cooled to solidify the agarose. From the solidified lobes, tissue cores (8 mm in diameter) containing a small vessel were prepared and sliced at a thickness of 350 µm using a VF300 vibratome (Precisionary, Greenville, NC). The slices were maintained in supplemented Ham’s F12 medium at 37 °C in a humidified air-CO_2_ (95–5%) incubator, with 3 medium changes during the 48 h. Adjacent slices containing contiguous segments of the same vessel served as controls for the experimental treatments. Following the wash period, the slices were incubated in normoxic (21% O_2_) or intermediate duration hypoxic (5% O_2_) conditions. To assess the involvement of NMDA receptor in regulation of vasoreactivity of pulmonary artery, slices were preincubated with MK801 (10 µM) for one hour and then vascular constriction were measured in the presence of varying concentrations of phenylephrine (10^–8^–10^–4^ M).

To measure vasoconstriction, the vessel in each slice was visualized under a microscope (Nikon Eclipse, × 40 magnification) and images were captured using an in-line camera (Evolution QEi, Silver Spring, MD). Luminal areas of the imaged vessels were quantified using Image-Pro Plus Software (version 6, Media Cybernetics, Silver Spring, MD) and represented in units of square micrometers. The luminal area at each concentration of phenylephrine was obtained and constriction of the vessels was calculated as a percentage of baseline luminal area. Unpaired Student’s t-tests were performed to determine statistical significance at the *p* < 0.05 confidence level when comparing different treatment groups.

To prepare murine precision cut lung slices, 8-week-old C57BL/6NJ mice (21.3 ± 1.8 g) were euthanized by intraperitoneal injection of sodium phenobarbital (250 mg/kg)^49^. Precision cut lung slices (Thickness: 200 µM) were prepared according to previously published methods^50^) with modifications^21^ relevant to vessels. Vasoconstriction was measured using procedures similar to that of human PCLS. Following pretreatment for one hour with MK801 (10 µM), AP5 (50 µM) or vehicle (1% DMSO), concentration–response curves of vascular constriction with increasing concentrations of endothelin-1 (ET-1) (10^–9^–10^–7^ M) were measured. Statistical differences were analyzed by one-way analysis of variance (ANOVA) test.

## Supplementary Information

Supplementary Information.

## Data Availability

All data generated or analysed during this study are included in this published article (and its Supplementary Information files).
