# Supplementary material for: Functional NMDA receptors are expressed by human pulmonary artery smooth muscle cells
Source: Sci Rep. 2021 Apr 15;11:8205. doi: 10.1038/s41598-021-87667-0 (PMC8050278; doi:10.1038/s41598-021-87667-0)
Supplement: Supplementary file 1 — Supplementary Information. [file 41598_2021_87667_MOESM1_ESM.docx]

**Supplementary information for**

**Functional NMDA receptors are expressed by human pulmonary artery smooth muscle cells**

Yi Na Dong^1^, Fu-Chun Hsu^1^, Cynthia J. Koziol-White^2^, Victoria Stepanova^3^, Joseph

Jude^2^, Andrei Gritsiuta^4^, Ryan Rue^4^, Rosalind Mott^5^, Douglas A. Coulter^1^, Reynold A.

Panettieri, Jr^2^, Vera P. Krymskaya^4^, Hajime Takano^1^, Elena A. Goncharova^6^, Dmitry A.

Goncharov^6^, Douglas B. Cines^3^, David R. Lynch^1^

David R Lynch

Email: [lynchd@pennmedicine.upenn.edu](mailto:lynchd@pennmedicine.upenn.edu)

Supplemental Table 1

Detailed information of Human lung tissue donors.

|  | **number** | **median age** | **mean age** | **age range** |
| --- | --- | --- | --- | --- |
| **Male** | 10 | 37.5 | 37.7 | 21-53 |
| **Female** | 3 | 34 | 37 | 22-55 |
| **Caucasian** | 7 |  |  |  |
| **African American** | 3 |  |  |  |
| **Hispanic** | 3 |  |  |  |

**Supplemental Figure 1.** *GluN2B mRNA expression in HPASMCs*. RT-PCR was performed for RNA isolated from HPASMCs. The presence of mRNA for GluN2B was detected in HPASMCs. Human brain RNA was used as a positive control.


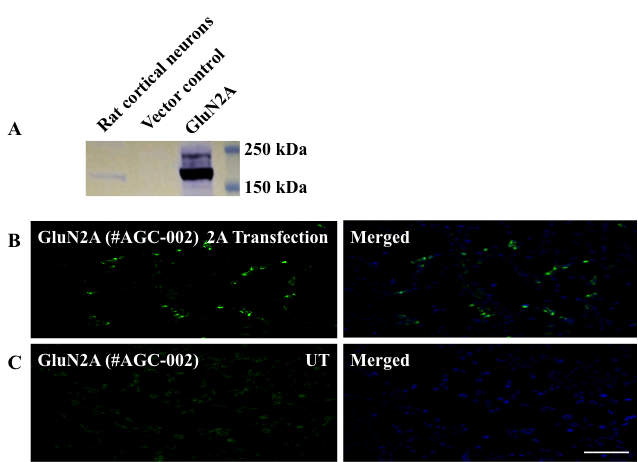


**Supplemental Figure 2**. Validation of the antibody against GluN2A. HEK 293 cells were transfected with GluN2A cDNA for 24 hours and then subjected to Western blot or immunocytochemistry with the indicated antibody. GluN2A antibody recognizes a 180 kDa protein from rat cortical neurons and in HEK 293 cells transfected with GluN2A. Vector control transfected cells show no signal (A). In immunocytochemistry, the antibody labeled HEK 293 cells transfected with GluN2A (B) while untransfected cells (UT) (C) remained unstained. Results are representative of three separate experiments. Scale bar=50 μM.


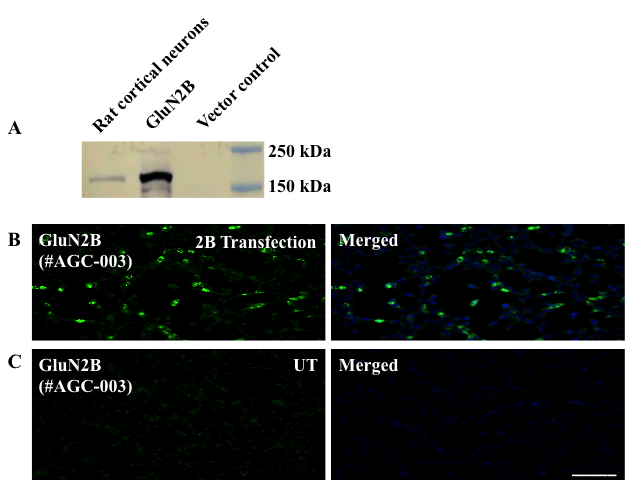


**Supplemental Figure 3**. Validation of the antibody against GluN2B. HEK 293 cells were transfected with GluN2B cDNA for 24 hours and then subjected to Western blot or immunocytochemistry with the indicated antibody. The antibody recognizes a 180 kDa protein from rat cortical neurons and in HEK 293 cells transfected with GluN2B. Vector control transfected cells show no signal (A). In immunocytochemistry, the antibody labeled HEK 293 cells transfected with GluN2B (B) while untransfected cells (UT) (C) remained unstained. Results are representative of three separate experiments. Scale bar=50 μM.


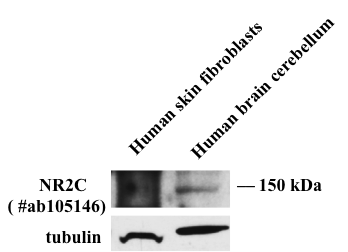


**Supplemental Figure 4**. Validation of the antibody against GluN2C. Lysates from human brain cerebellum or human skin fibroblasts were subjected to Western blot with the indicated antibody. The antibody recognizes a 150 kDa protein from human brain cerebellum but not human skin fibroblasts. Results are representative of three separate experiments.


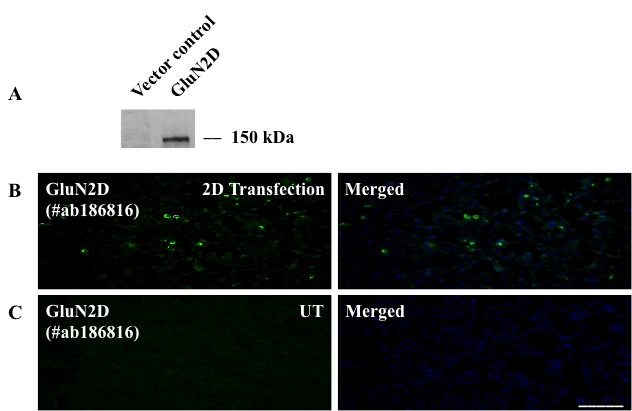


**Supplemental Figure 5**. Validation of the antibody against GluN2D. HEK 293 cells were transfected with GluN2D cDNA for 24 hours and then subjected to Western blot or immunocytochemistry with the indicated antibody. The antibody recognizes a 150 kDa protein in HEK 293 cells transfected with GluN2D. Vector control transfected cells show no signal (A). In immunocytochemistry, the antibody labeled HEK 293 cells transfected with GluN2D (B) while untransfected cells (UT) (C) remained unstained. Results are representative of three separate experiments. Scale bar=50 μM.


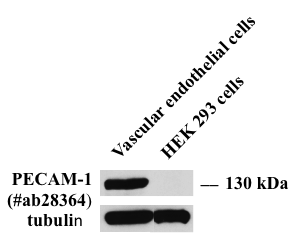


**Supplemental Figure 6**. Validation of the antibody against PECAM. Lysates from human vascular endothelial cells or HEK 293 cells were subjected to Western blot with antibody against PECAM. The antibody recognizes a 130 kDa protein from human vascular endothelial cells but not HEK 293 cells. Results are representative of three separate experiments.


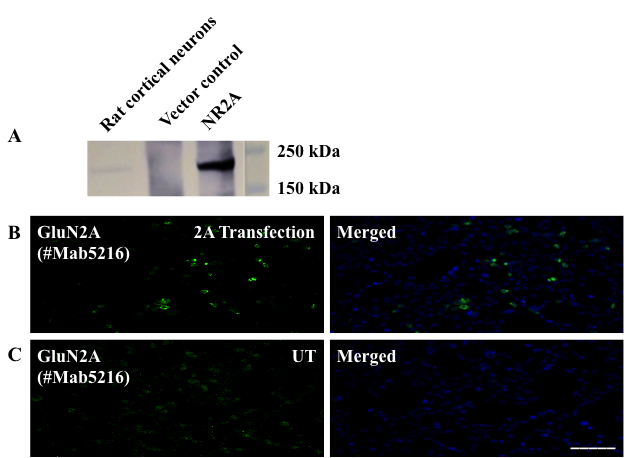


**Supplemental Figure 7**. Validation of the antibody against GluN2A. HEK 293 cells were transfected with GluN2A cDNA for 24 hours and then subjected to Western blot or immunocytochemistry with the indicated antibody. The antibody recognizes a 180 kDa protein from rat cortical neurons and in HEK 293 cells transfected with GluN2A. Vector control transfected cells show no signal (A). In immunocytochemistry, the antibody labeled HEK 293 cells transfected with GluN2A (B) while untransfected cells (UT) (C) remained unstained. Results are representative of three separate experiments. Scale bar=50 μM.


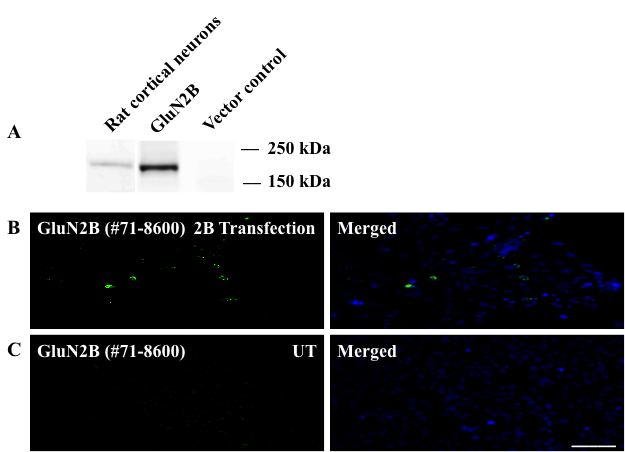


**Supplemental Figure 8**. Validation of the antibody against GluN2B. HEK 293 cells were transfected with GluN2B cDNA for 24 hours and then subjected to Western blot or immunocytochemistry with the indicated antibody. The antibody recognizes a 180 kDa protein from rat cortical neurons and in HEK 293 cells transfected with GluN2B. Vector control transfected cells show no signal (A). In immunocytochemistry, the antibody labeled HEK 293 cells transfected with GluN2B (B) while untransfected cells (UT) (C) remained unstained. Results are representative of three separate experiments. Scale bar=50 μM.


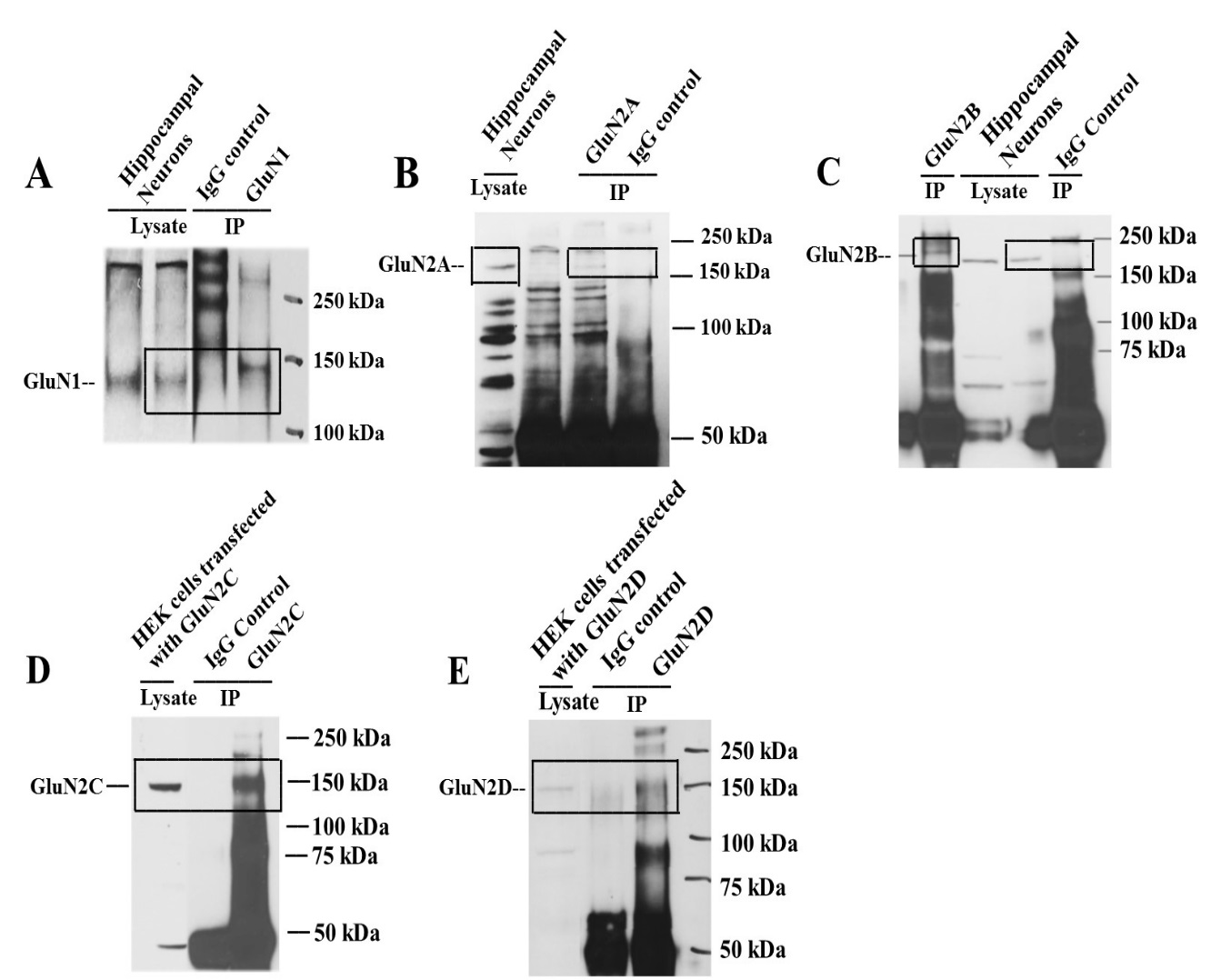


**Supplemental Figure 9**. Uncropped Western blots for Immunoprecipitated NMDA receptors in HPASMCs by subunit specific antibodies or control non-immune IgG (A: GluN1; B: GluN2A; C: GluN2B; D: GluN2C; E: GluN2E). The black box marks the samples that are presented in Figure 4.


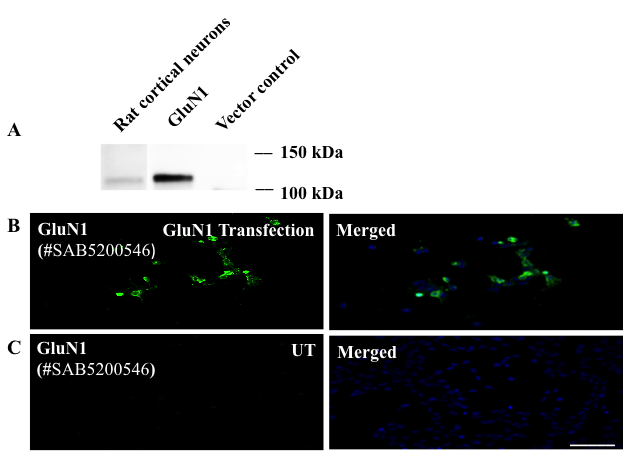


**Supplemental Figure 10.** Validation of the antibody against GluN1. HEK 293 cells were transfected with GluN1 cDNA for 24 hours and then subjected to Western blot or immunocytochemistry with the indicated antibody. The antibody recognizes a 120 kDa protein from rat cortical neurons and in HEK 293 cells transfected with GluN2A. Vector control transfected cells show no signal (A). In immunocytochemistry, the antibody labeled HEK 293 cells transfected with GluN1 (B) while untransfected cells (UT) (C) remained unstained. Results are representative of three separate experiments. Scale bar=50 μM.


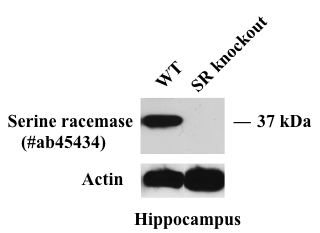


**Supplemental Figure 11**. Validation of the antibody against serine racemase. Hippocampal lysates from wild-type or serine racemase knockout mouse were subjected to Western blot with the indicated antibody. The antibody recognizes a 37 kDa protein in hippocampal lysates from wild-type but not serine racemase knockout mouse. Results are representative of three separate experiments.


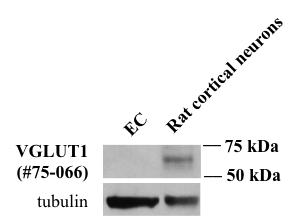


**Supplemental Figure 12**. Validation of the antibody against VGLUT1. Lysates from human vascular endothelial cells or rat cortical neurons were subjected to Western blot with antibody against VGLUT1. The antibody recognizes a 62 kDa protein from rat cortical neurons but not human vascular endothelial cells. Results are representative of three separate experiments.

**Supplemental Figure 13**. Uncropped Western blots of serine racemase (A: 37 kDa) and VGLUT1 (B: 62 kDa). The black box marks the samples that are presented in Figure 6.
